# 3D virtual surgical planning of fractures in hip arthrodesis: a systematic review, case series and recommendations for treatment

**DOI:** 10.1007/s00590-025-04283-8

**Published:** 2025-04-12

**Authors:** Raul G. Plomp, Kaj Ten Duis, Anne M. L. Meesters, Frank F. A. IJpma

**Affiliations:** 1 Department of Trauma Surgery, University Medical Center Groningen, Groningen, The Netherlands; 2 3D Lab, University Medical Center Groningen, Groningen, The Netherlands

**Keywords:** Hip arthrodesis fracture, Ankylosis, 3D, Systematic review

## Abstract

**Purpose:**

Fractures through an arthrodesed hip are rare and challenging. The aim of the study is (1) to explore whether 3D-planned percutaneous screw fixation of fractures in hip arthrodesis is a viable minimally invasive surgical option for geriatric patients and (2) to standardize surgical treatment by providing a comprehensive overview of the literature and propose a treatment algorithm.

**Methods:**

We presented a case series of patients with an acute fracture in a previous hip arthrodesis treated in a level 1 trauma centre in 2024. Furthermore, we conducted a systematic review on fractures in hip arthrodesis from 1970 to 2023.

**Results:**

We presented three cases treated for a fracture in an arthrodesed hip. Two patients with a proximal/medial fracture to the acetabulum were operated with 3D-planned percutaneous cannulated screws, and one patient with an intertrochanteric fracture was operated with a DHS system. The systematic review resulted in an overview of 16 case series on fractures in hip arthrodesis treated with various surgical techniques, each with its pros and cons; cannulated screws, DHS system, intramedullary nailing and plate osteosynthesis.

**Conclusion:**

Acute fractures in arthrodesed hips in fragile geriatric patients can be treated minimally invasively with 3D-planned percutaneous screw fixation. This technique is most suitable for femoral neck fracture types. Alternative surgical techniques include DHS, intramedullary nailing, plate osteosynthesis or conversion to total hip arthroplasty, for which a treatment algorithm is provided.

## Introduction

Hip arthrodesis can serve as a salvage procedure for patients with unilateral isolated osteoarthritis, severe muscular deficits or painful chronic hip conditions, particularly posttraumatic ones [1, 2]. In these conditions, total hip arthroplasty is not always a solution due to its high failure rates, with multiple revisions reported in up to 45% of cases [2]. Fortunately, there are few patients with hip arthrodesis, and fractures in an arthrodesed hip have only been reported in a few cases worldwide [3]. A long-term follow-up of hip arthrodesis patients in 1984 showed that only two of 53 (4%) patients sustained a femoral fracture [4]. Among patients with a hip arthrodesis, intertrochanteric fractures are most frequently observed, while femoral neck fractures are less common [5].

The altered anatomy and the significant forces acting on a fractured arthrodesed hip make surgery challenging. Additionally, due to its rarity and the lack of literature on these fractures, there is no consensus or definitive treatment strategy. Font et al. used cannulated hip screws for an intertrochanteric fracture in an ankylosed hip in 2010 [6]. Ever since, a variety of treatment modalities have been described, of which invasive plating osteosyntheses are the most commonly used [3, 6–17]. It is known that 3D-assisted surgery could reduce the operation time, intraoperative blood loss, fluoroscopy usage and complications in acetabular surgery [18]. However, only one study has explored the use of conventional plating combined with a 3D-printed model for fracture reconstruction in hip arthrodesis [19]. The clinical relevance of the current study is that most of these patients are fragile elderly, for whom major procedures pose risks, making it worthwhile to explore minimally invasive treatment options.

Therefore, the aim of the study is (1) to explore whether 3D-planned percutaneous screw fixation of fractures in hip arthrodesis is feasible, and (2) to standardize the surgical treatment by providing a comprehensive overview of the literature and propose a treatment algorithm.

## Materials and methods

### Study population

We present a case series of patients with fractures in hip arthrodesis treated in a level 1 trauma centre. Patients with an acute fracture in a previous hip arthrodesis were included.

### 3D virtual surgical planning

A virtual 3D model was created from the CT scan of each patient using the Mimics Medical 19.0 software (Materialize, Leuven, Belgium). The model was created based on the Hounsfield Units, and the fractured part of the hip was separated from the rest of the pelvis. Next, the fracture was virtually reduced. The screw trajectories were planned using a cylinder with a diameter of 6.5 mm.

### Systematic review

A systematic search was performed in PubMed. The search terms “hip arthrodesis” and “fracture” were used. The years searched were from 1st January 1970 until 31st December 2023. All study types were eligible for inclusion. All articles were screened for eligibility based on titles and abstract by the first, second and last author. The same reviewers independently conducted a full-text screening of the remaining articles. Finally, the references of the included articles were screened for additional relevant articles.

## Results

Recently, three patients with a fractured hip arthrodesis were referred and treated at our tertiary trauma university medical centre. All three cases had a history of hip arthrodesis and presented with a recent fracture at the site.

### Case 1

**Fig. 1:**
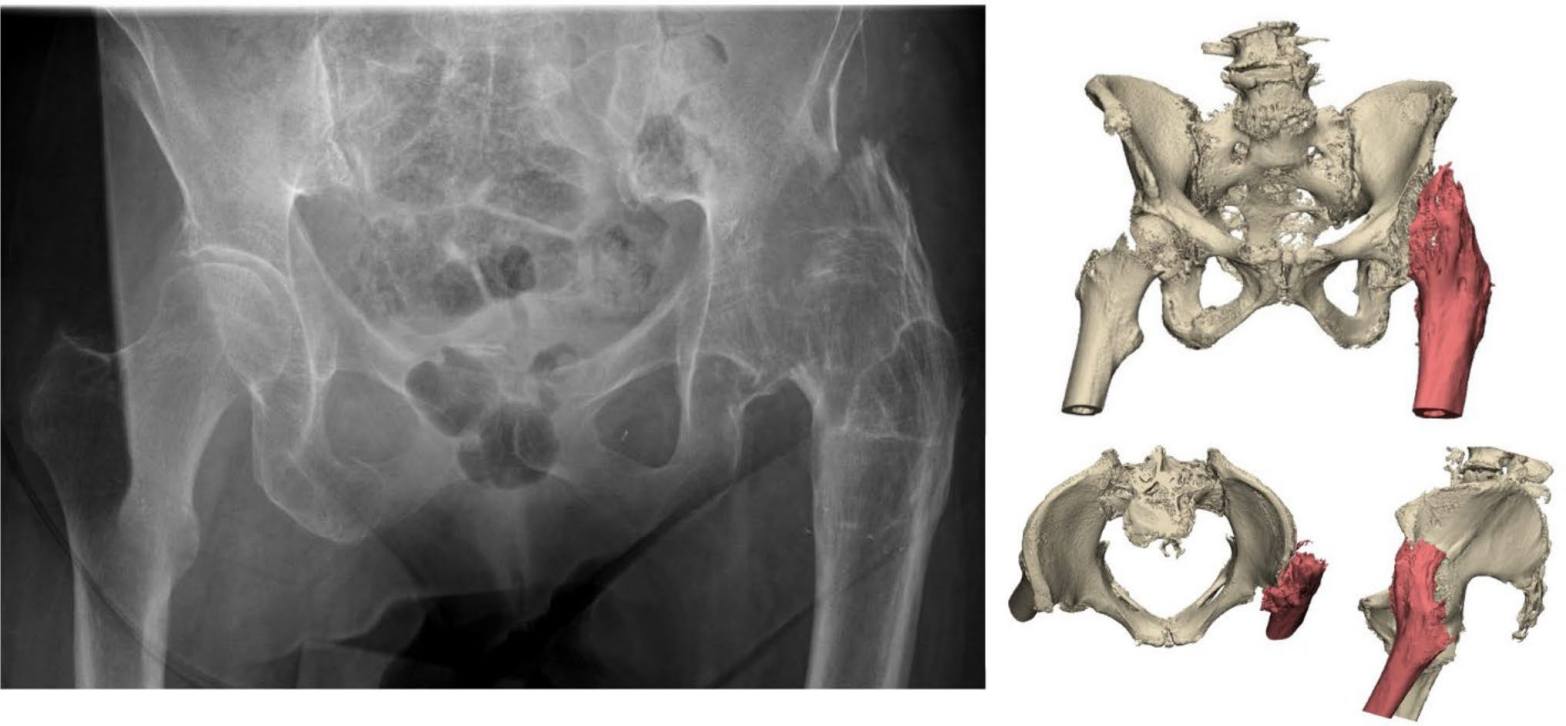
**Case 1.** Preoperative X-ray and 3D reconstruction, a fracture at the level of the pelvis/acetabulum of the left arthrodesed hip

**Fig. 2:**
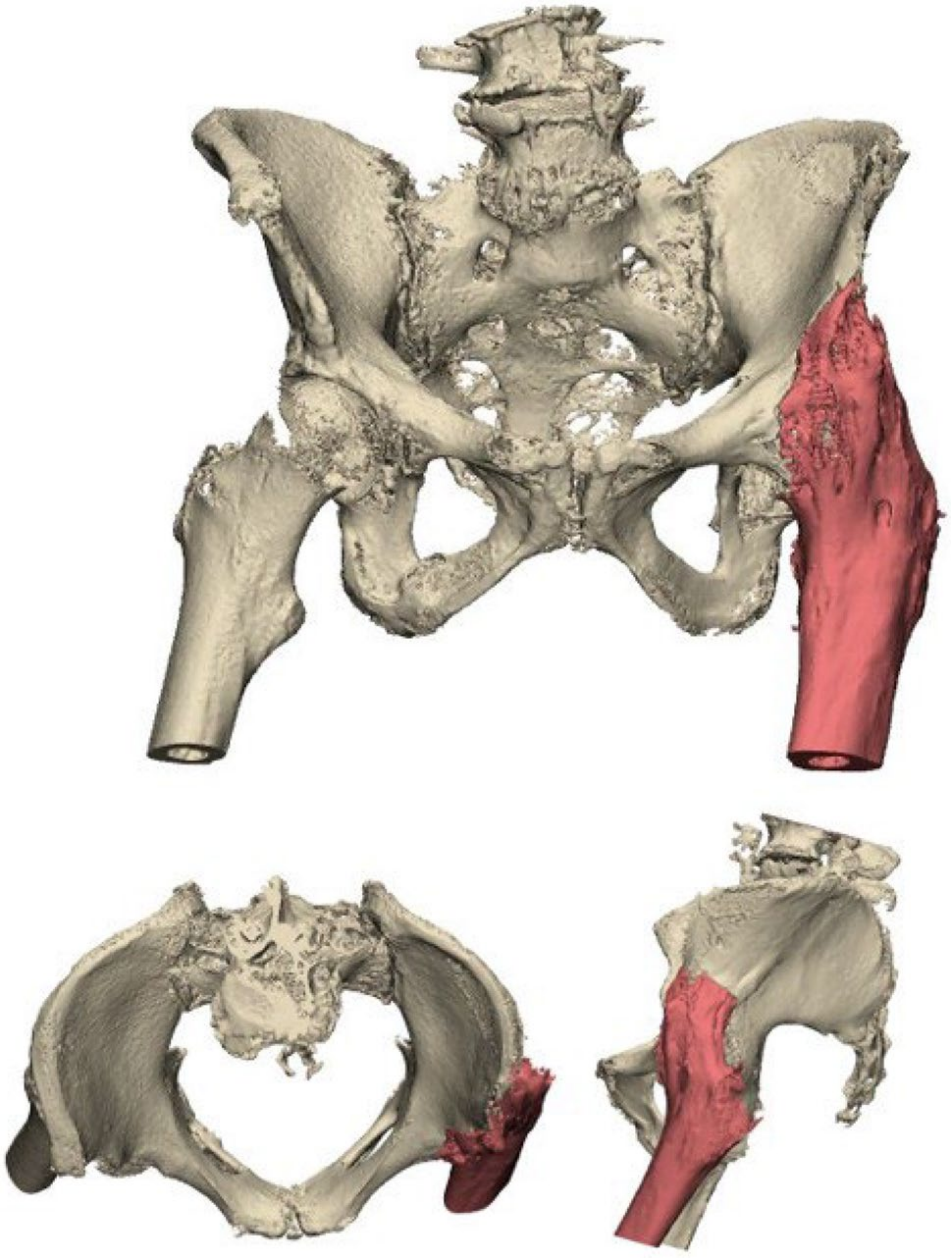
**Case 1.** 3D reconstruction with reduction of the fracture of the hip arthrodesis

**Fig. 3:**
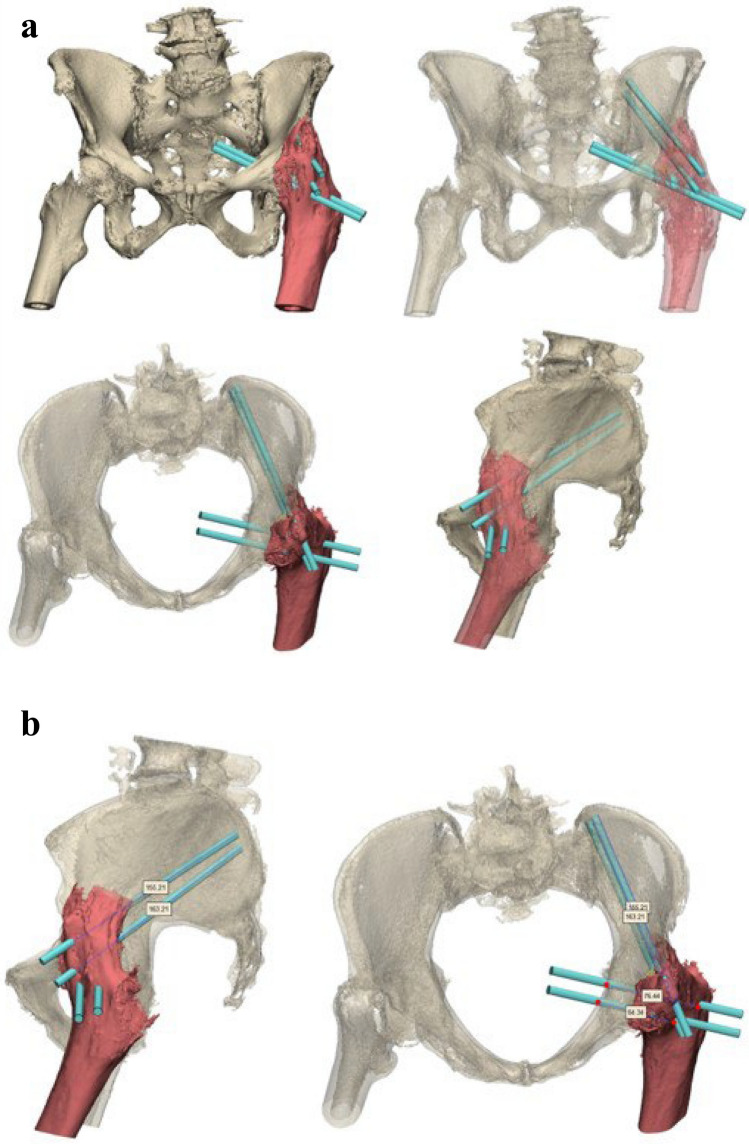
**a** **Case 1.** 3D preoperative surgical planning of percutaneous cannulated screw fixation. **b**. **Case 1.** 3D preoperative surgical planning of percutaneous cannulated screw fixation

**Fig. 4:**
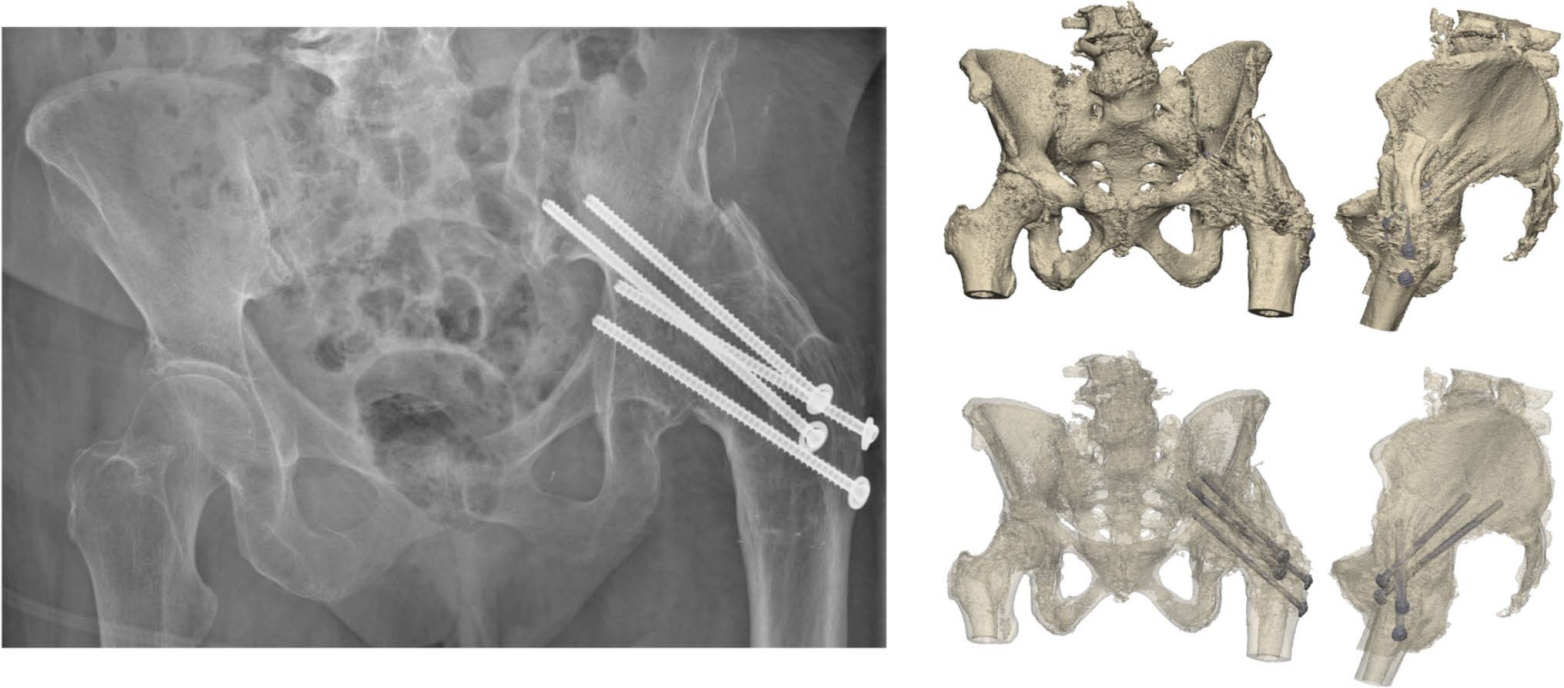
**Case 1.** Postoperative result

An 86-year-old woman was referred after a fall on her left hip. She was able to walk short distances with a walking aid. Around 45 years ago, she underwent an arthrodesis of the left hip due to Perthes’ disease. Following this, she was treated with a cast for the duration of three months. Her medical history also includes a total knee arthroplasty on the right side and a cerebrovascular accident, from which she recovered uneventfully. Radiographs and 3D reconstruction of the CT scan demonstrated a medial fracture of the hip arthrodesis at the level of the acetabulum (Fig. 1). The 3D virtual fracture model was created to simulate the reduction of the fracture (Fig. 2) and to plan the percutaneous cannulated screw trajectories (Fig. 3a, b). The patient was operated on in a supine position on a table with the leg in traction. Closed reduction was achieved with traction and endorotation of the leg. Four minimally invasive percutaneously placed cannulated screws (diameter 6.5 mm) were inserted as planned, including two LC-2 screws. The operation time was 1 h 26 min, and the blood loss was negligible. The postoperative course was uncomplicated, and after seven days, she was discharged to a nursing home. Due to her advanced age, her mobility was limited, and she used a walking frame to move from bed to chair. At twelve weeks postoperatively, she immediately began full weight-bearing, and she regained her pre-injury level of functioning with progressive fracture healing visible on the pelvic X-ray (Fig. 4).

### Case 2

**Fig. 5:**
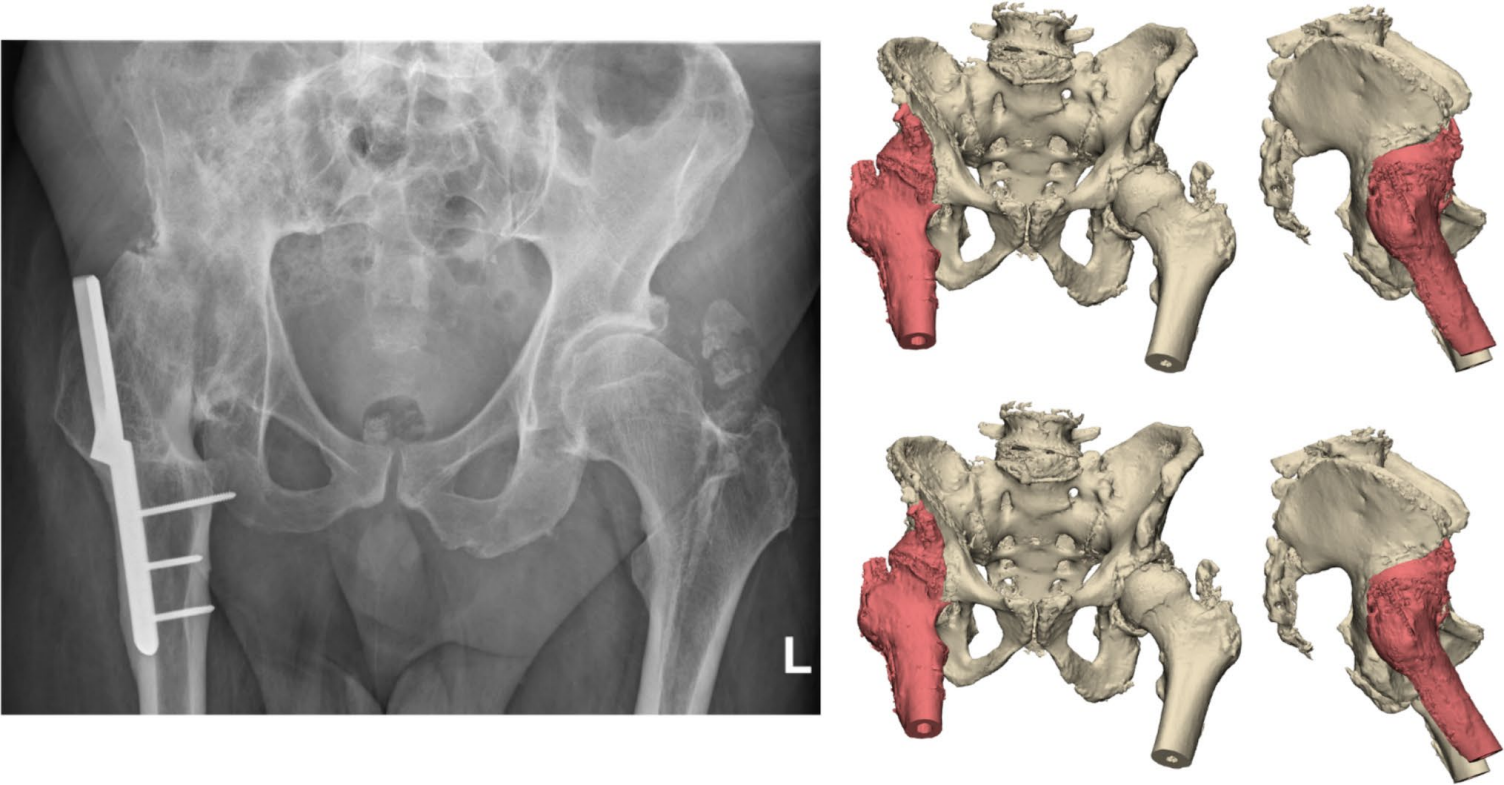
**Case 2**. Preoperative X-ray and 3D reconstruction and reduction of the fracture of the right hip arthrodesis

**Fig. 6:**
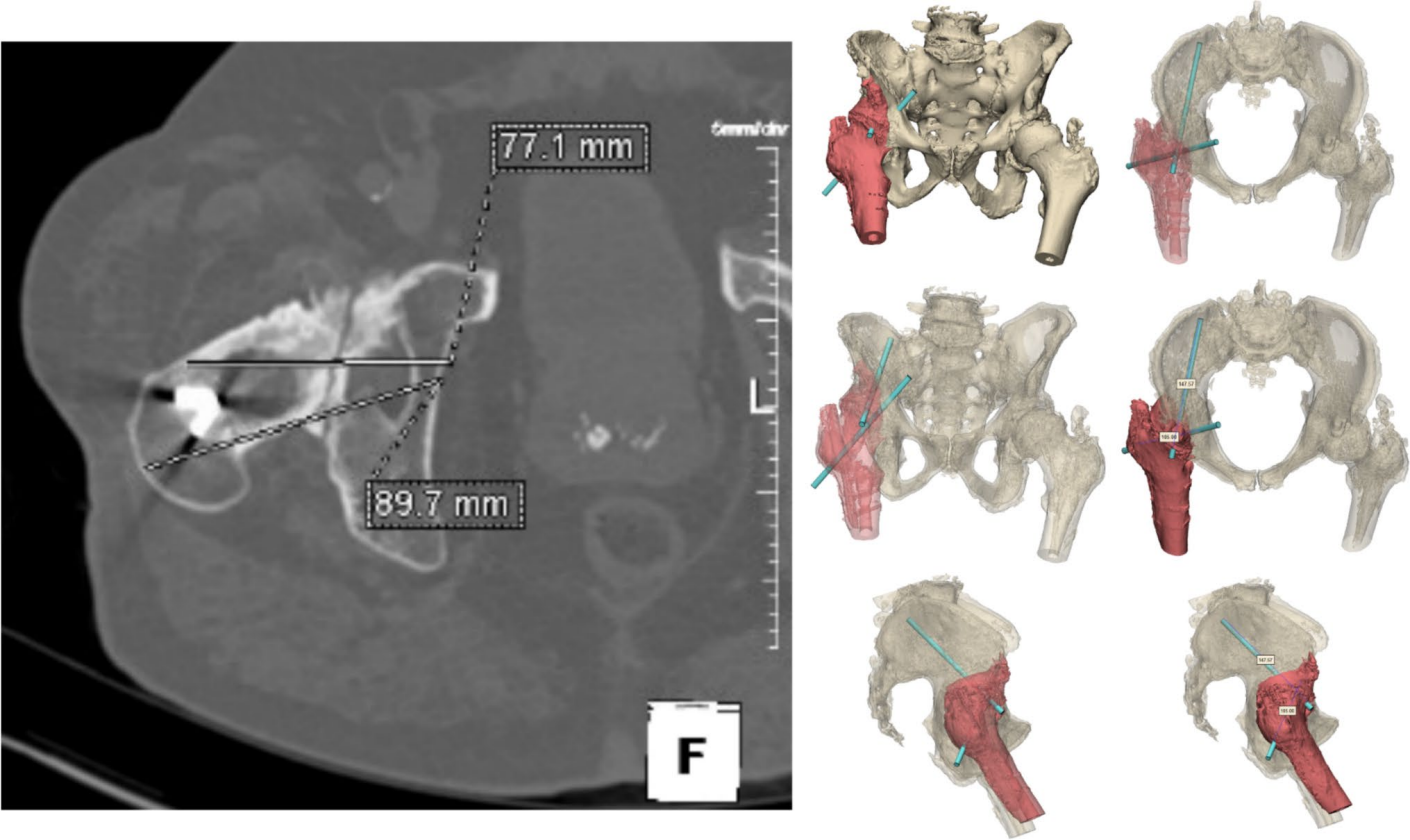
**Case 2.** Preoperative CT and 3D planning of percutaneous minimal invasive screws around the already existing plate

**Fig. 7:**
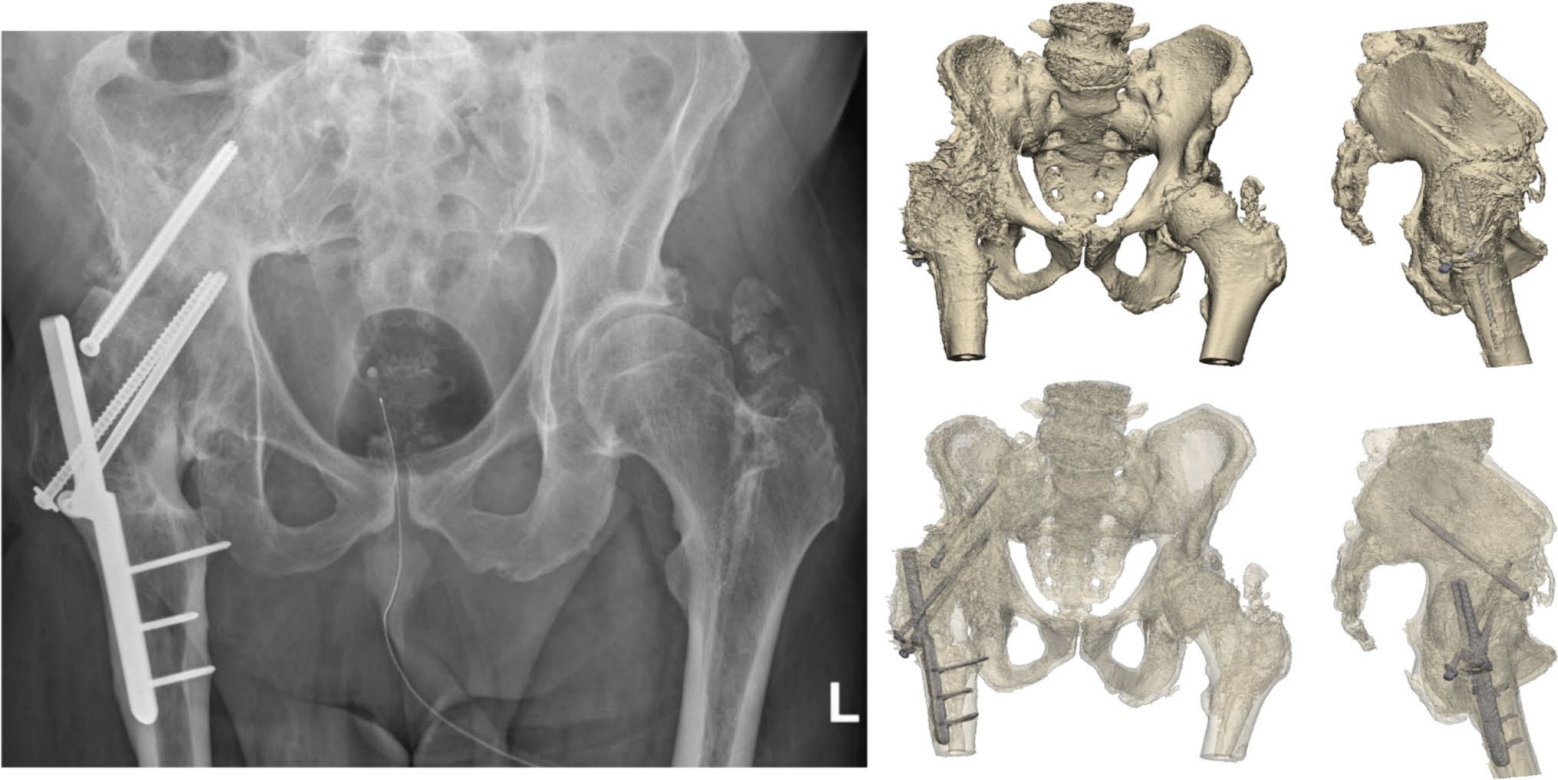
**Case 2.** Postoperative result of minimal invasive percutaneous cannulated screws

A 76-year-old man was referred from a regional hospital after a fall on his right hip. He had a medical history of atrial fibrillation and coronary artery bypass grafting. At the age of 21 years, he underwent a plate osteosynthesis, corrective osteotomy of the proximal femur and developed a fracture-related infection, which resulted in an arthrodesis of the hip. Before the injury, he used a walking aid, and he had an action radius of 1 km. The radiograph and 3D reconstruction of the CT scan revealed a highly proximal/medial fracture of the right hip arthrodesis, extending almost into the pelvis/acetabulum (Fig. 5). The patient was operated on in a supine position on a traction table with the leg in traction. Closed reduction was performed, and the fracture was fixed with three percutaneous (6.5 mm) screws. Two screws were placed on both sides (i.e. anterior and posterior) of the in situ plate (Fig. 6). One cannulated screw (i.e. LC-2 or teardrop screw) was placed by using an obturator outlet view with fluoroscopy (Fig. 7). The operation time was 1 h 33 min, and there was the blood loss was negligible. The postoperative course was complicated by delirium. Twelve days after the surgery, the patient was discharged to a nursing home. Eight weeks postoperatively, he began 50% weight-bearing. The follow-up radiographs showed progressive fracture healing.

### Case 3

**Fig. 8:**
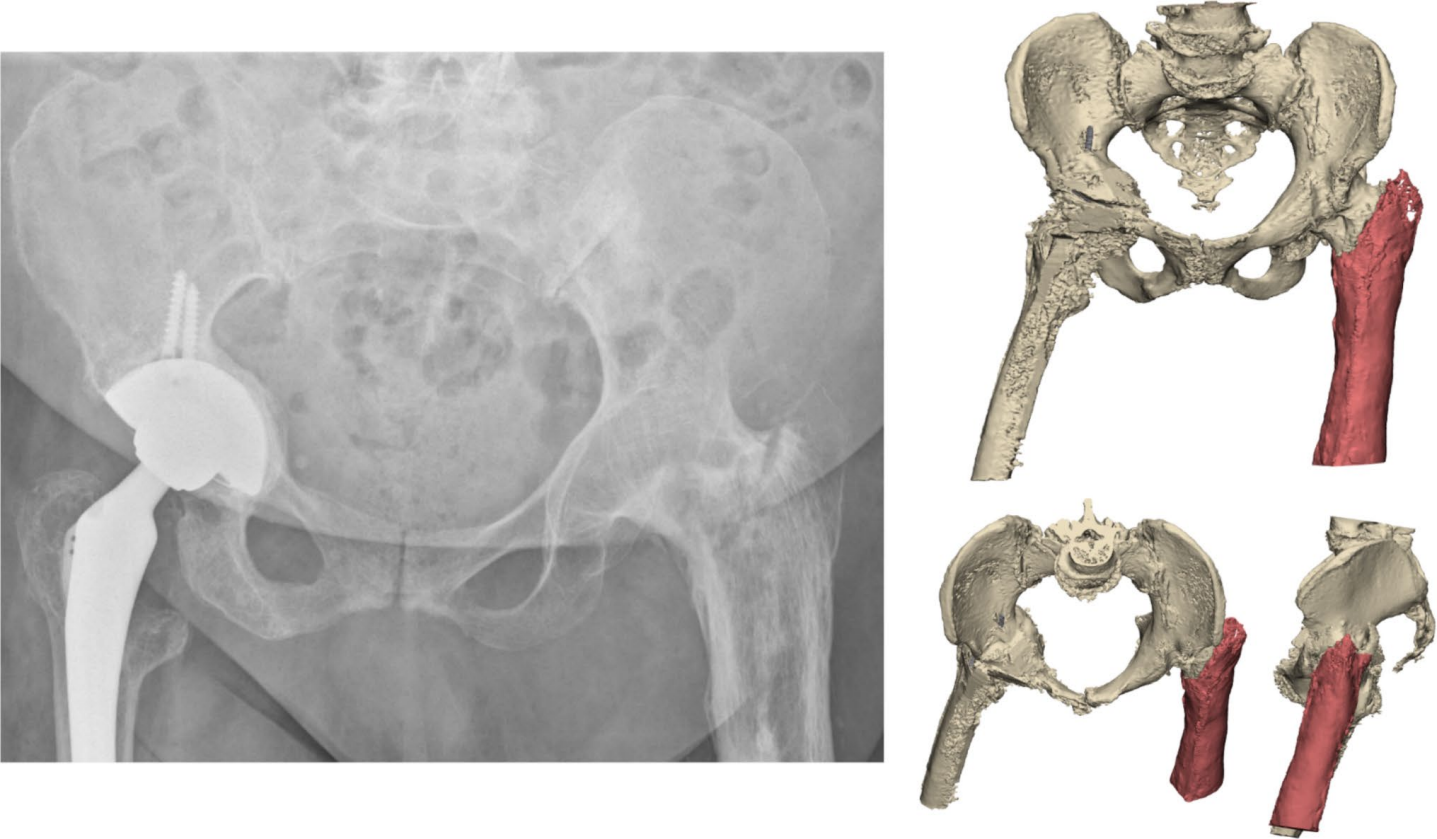
**Case 3.** Preoperative X-ray and 3D reconstruction of left intertrochanteric/lateral collum fracture in a an arthrodesed hip

**Fig. 9:**
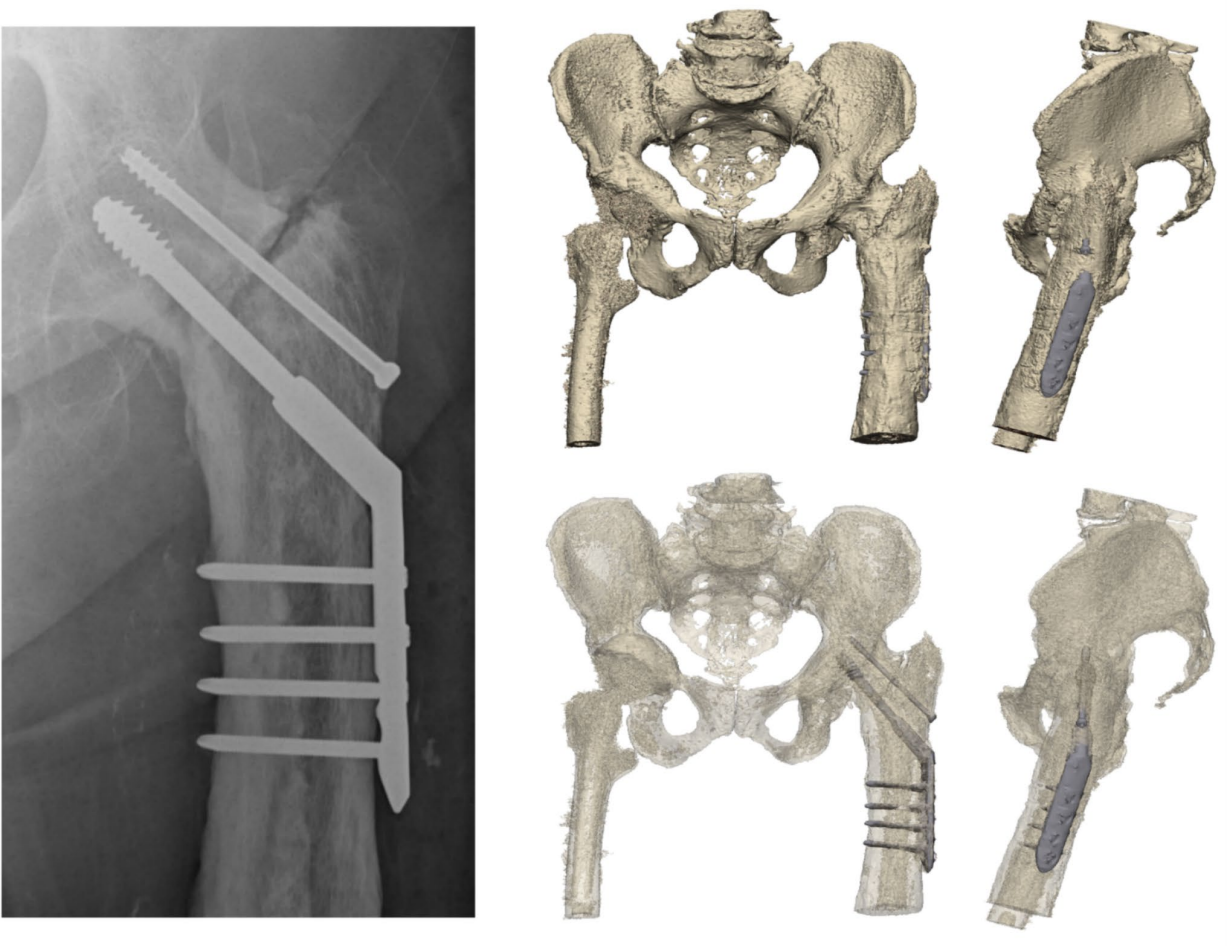
**Case 3.** Postoperative result of a dynamic hip screw augmented with a cannulated screw in a fracture in an arthrodesed hip

**Fig. 10:**
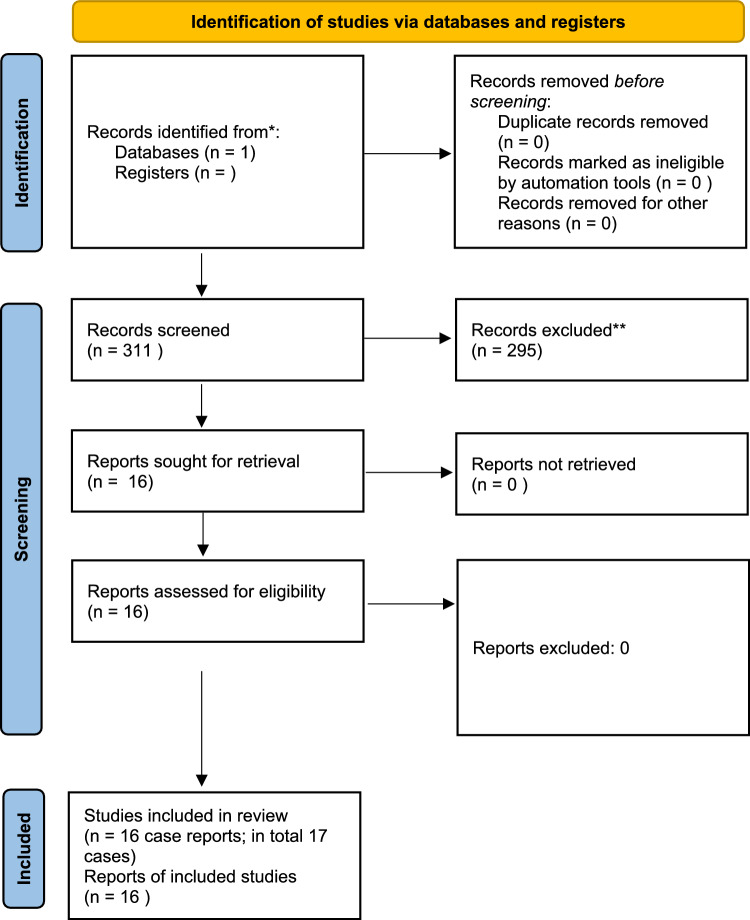
Flowchart of the systematic literature search

An 80-year-old woman was referred after a fall onto her left hip. Her medical history included of total hip arthroplasty on the right side and hip arthrodesis following severe septic arthritis on the left side in 1958. Additionally, a knee arthrodesis was performed on the left side in 2008 following osteomyelitis of the knee. He was able to walk short distances with a walking aid. The radiograph and 3D CT scan demonstrated an intertrochanteric/lateral column fracture of the left hip arthrodesis (Fig. 8). The patient was operated on in a supine position on a traction table. A four-hole dynamic hip screw (DHS, DePuy Synthes) and a cannulated screw were used to stabilize the fracture (Fig. 9). Operation time was 1h 12 min, with minimal blood loss. The postoperative course was uncomplicated, and the patient was discharged to a rehabilitation centre after four days (Fig. 10).

### Systematic review

A total of 311 studies were identified, of which 16 were considered eligible after screening titles and abstracts. All included studies were case reports, with a total of 17 patients (Table 1).

**Table 1:** Overview results, case reports and surgical treatment of fractured hip arthrodesis

Author	Year	Fracture type	Age	M/F*	Treatment	Approach	Results	Weight-bearing	Healing time
Andreas	2004	Femoral neck fracture	86	F	Retrograde supracondylar intramedullary nail inserted from trochanteric area into the ileum	Minimal invasive and screws via groyne	Satisfactory	Full weight-bearing after 1 week	3 months
Manzotti	2007	Intertrochanteric fracture	74	F	Double-plating anterior and lateral	Not described	Satisfactory	Partial weight-bearing; after 7 weeks full weight-bearing	19 months
Font	2010	Intertrochanteric fracture	68	F	Four screws (7.0 mm) for fixation towards acetabular columns	Direct lateral approach small incision	Satisfactory	Non weight-bearing 6 weeks	1 year
Ishimaru	2012	Intertrochanteric fracture in ankylosed hip after failed arthrodesis	76	M	Proximal femoral nail (gamma nail)	Minimal invasive	Satisfactory	Partial weight-bearing at 2 weeks after surgery	1 year
Darwish	2013	Intertrochanteric fracture	30	F	Anterolateral locked plate and cannulated screws	Direct lateral approach	Satisfactory	Walking with crutches day 1	4 months
Fang	2015	Intertrochanteric fracture	88	F	Spanning reversed distal femur locking plate (MIPO) in addition to in situ DHS**	Minimal invasive	Satisfactory	Quadripod full weight-bearing	4 months
Asakawa	2017	Proximal femoral neck fracture	90	F	Double plate fixation (posterior and anterior column)	Direct lateral	Satisfactory	4 weeks hip spica postop, then partially weight-bearing	3 months
Delanu	2017	Intertrochanteric fracture	62	F	Removal cannulated screws (arthrodesis) Lateral locking plate	Direct lateral	Satisfactory	After 2 weeks weight-bearing double crutches	3 months
Pascarella	2018	Subtrochanteric fracture	61	F	Lateral locking plate after failed intramedullary nailing after failed locking plate (non-union)	Not described	Satisfactory	No weight-bearing 2 months	6 months
Nelson	2019	Intertrochanteric fracture	81	M	LC-DCP 4.5 plate anterolateral + DHS	Direct lateral	Satisfactory	Hip abduction brace, toe-touch weight-bearing 3 months	3 months
Devkota	2019	Intertrochanteric fracture	74	M	DHS + cannulated screw	Direct lateral	Satisfactory	Non weight-bearing 6 weeks, partial weight-bearing 6–12 weeks, full weight-bearing after 12 weeks	18 months
Pato	2020	Intertrochanteric fracture	83	F	Lateral locking femoral plate, with + cannulated screws + 3.5 mm pelvic reconstruction plate	Direct lateral	Satisfactory	No weight-bearing first 3 weeks, partially weight-bearing till 3 months	3 months
Pogliacomi	2020	Subtrochanteric fracture	71	F	DHS with platelet-rich plasma after failed (non-union at 8 months) lateral plate	Not described	Revision surgery	Non weight-bearing 2 months	8 months
Malhotra	2020	Intertrochanteric fracture (non-union of a DCS*** plate)	68	M	Acute conversion to total hip arthroplasty	Posterior	Satisfactory	Toe-touch weight-bearing 6 weeks	6 months
		Subtrochanteric fracture	42	F	Acute conversion to total hip arthroplasty	Posterior	Satisfactory	Full weight-bearing day 1	12 months
Cardile	2021	Intertrochanteric fracture	73	F	Lateral 4, 5 condylar femur plate (contralateral upside down)	Not described	Satisfactory	Non weight-bearing 6 weeks, then partial weight-bearing, 2 months total weight-bearing	6 months
Bouzid	2023	Intertrochanteric fracture	45	M	PFNA****	Minimal invasive	Satisfactory	Partial weight-bearing 6 weeks, full weight-bearing after 6 weeks	8 months

*M/*F* male/female

**DHS dynamic hip screw

***DCS dynamic condylar screw

****PFNA proximal femoral nail antirotation

Five out of 17 patients were male (29%). The median age was 73 years (range 30–90). Patients experienced fractures at various levels of the arthrodesed hip, and twelve out of 17 patients sustained an intertrochanteric fracture, three a subtrochanteric fracture and two a femoral neck fracture. Nine patients were treated with plate osteosyntheses, of which two contained double plating. Three patients were treated with a DHS, of which one was in combination with a cannulated screw and one in combination with a plate. Three patients in total were treated with an intramedullary nail. One patient was treated with cannulated screws only.

In the cases of the double plating, the first plate was fixed to the anterior aspect of the femoral neck and the anterior column of the acetabulum, and the second plate was fixed to the lateral part of the greater trochanter and the superior aspect of the acetabulum. The main considerations in this case were the desire for fixation to the pelvis and a high femoral neck-shaft angle. The latter made a DHS or intramedullary nail impossible. The approach was not described [13]. In the other case of double plating, both the anterior column and the posterior column were stabilized [10].

Another case involved a lateral approach with a lateral locked plate and cannulated screws [14].

In general, the direct lateral approach was the most frequently used approach in seven cases, in four cases the approach was not described, and in four cases the approach was minimally invasive (one gamma nail, one PFNA, one retrograde supracondylar nail and one MIPO plating, two cases posterior approach for an acute conversion to a total hip arthroplasty) (Table 1).

All postoperative results were satisfactory from the index surgery, except for one case of plate osteosynthesis for a subtrochanteric fracture, which required revision surgery with a sliding hip screw (Table 1).

## Discussion

**Fig. 11:**
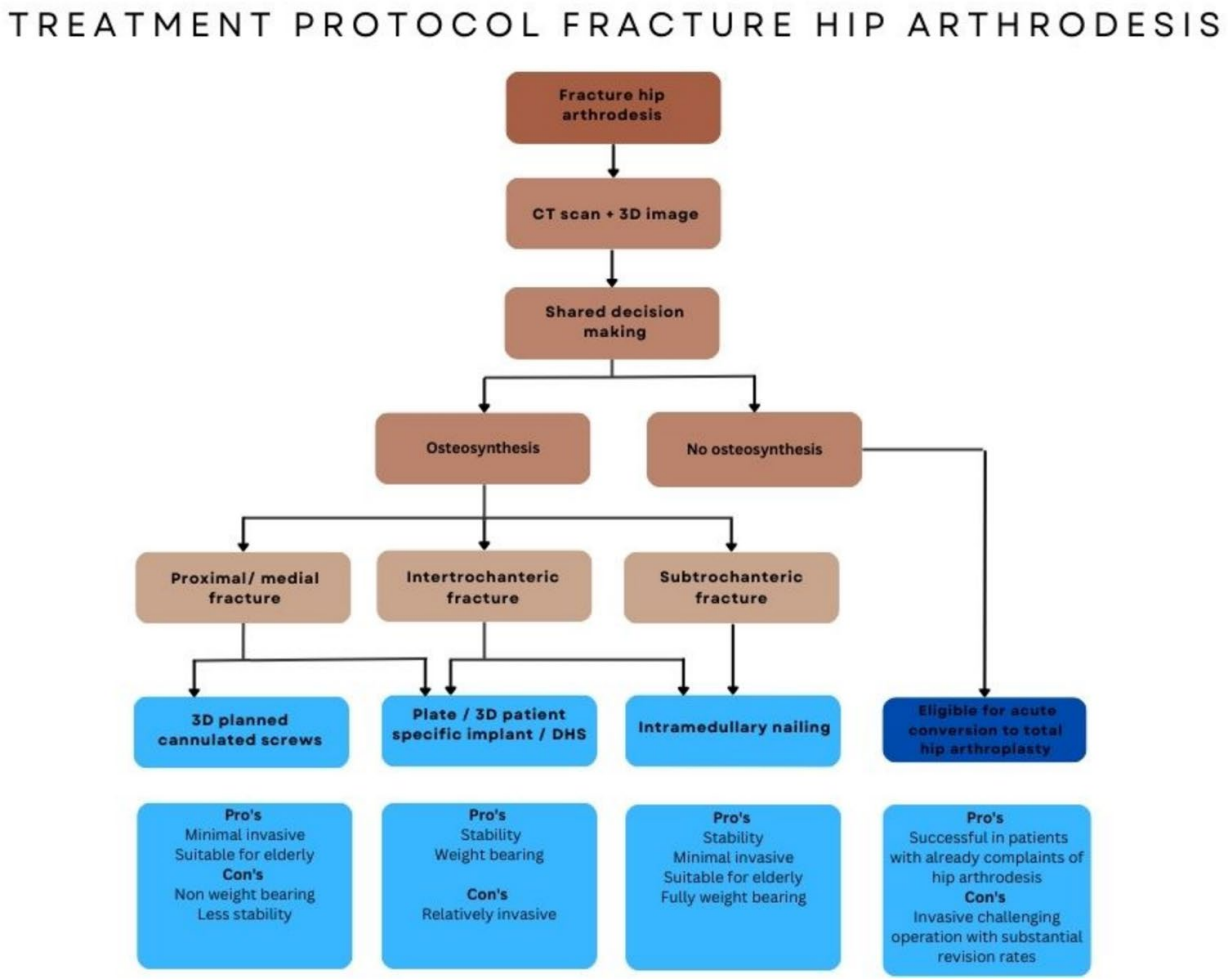
Flowchart fracture arthrodesed hips

In fragile geriatric patients, treatment of acute fractures in arthrodesed hips with minimally invasive 3D-planned percutaneous screw fixation is feasible. This case series and systematic review show that a variety of surgical techniques are used to treat acute fractures in hip arthrodesis, including cannulated screws, DHS system, plate osteosynthesis, intramedullary nailing or conversion to total hip arthroplasty. There is no convincing evidence for a specific implant choice other than described in case reports. We presented two cases treated with minimally invasive percutaneous 3D-planned cannulated screws and another case treated with a DHS. Based on our experiences, information from case series and fracture location, a treatment algorithm is proposed (Fig. 11).

*Cannulated screws* are commonly used to treat non-displaced femoral neck fractures with good results [20]. However, only one case in the literature reported the use of primarily cannulated screws for fractures in hip arthrodesis, and it was successful [6]. 3D virtual fracture visualization gives surgeons a better understanding of a fracture pattern [21, 22]. Moreover, 3D virtual fracture visualization and preoperative planning have an added value in minimizing the risk of screw malposition [23]. Our series adds to previous literature because in two cases (case one and two), we presented the use of 3D surgical planning of cannulated screw fixation of the fracture in the arthrodesed hip. We believe there are three main advantages: (1) exact preoperative screw path planning in these rare fracture types, especially when other osteosynthesis material is already in situ, (2) minimally invasive surgery instead of extensive exploration of the hip arthrodesis in the geriatric patient and (3) relatively short operation time and minimal blood loss. In the two cases treated with cannulated screws, we opted for non-weight-bearing due to of the long lever arm created by the fusion of the hip. We acknowledge this could be a disadvantage in the early postoperative active mobilization and rehabilitation.

*A DHS system* was used successfully in the third case. This concerned a more distal/lateral fracture type (intertrochanteric level). The advantage of a DHS is a limited surgical exposure, away from the compromised joint. In this specific case, intramedullary nailing was not possible due to the ipsilateral knee arthrodesis nail. We used a DHS system for a non-displaced intertrochanteric fracture. A DHS has been used only three times in the literature, often augmented with plate osteosyntheses. However, it remains debatable if this augmentation is truly necessary [9, 15, 24]. Also, there is some evidence that a DHS is feasible in intertrochanteric fractures in patients with severe pre-existent coxarthrosis/osteoarthritis (grade III and IV). While total hip replacement is usually indicated in this group, many patients regained mobility after the DHS, which is a less invasive procedure with a lower complication risk than *acute* total hip arthroplasty. This supports the hypothesis that primarily osteosynthesis with a feasible implant could have an acceptable outcome even in severely compromised hip joints [25]. A recent meta-analysis found a higher rate of refracture and reoperation in the general population with intertrochanteric fractures, favouring DHS over intramedullary nailing, suggesting that a DHS as an option in arthrodesed hip fractures should not be overlooked [26].

*Intramedullary nailing* has been described as a successful treatment for more lateral fractures, such as intertrochanteric fractures [8, 12, 27]. In the literature on fractures in hip arthrodesis, only three reports of nails were found: one used for a medial (neck) fracture (retrograde) and the other two for intertrochanteric fractures. The advantage of nailing, in general, is the minimally invasive approach and a disadvantage in this specific anatomy could be a more “ectopic” trochanter entry point. In our opinion, the minimally invasive approach could be advantageous in the geriatric population suffering from fractures in hip arthrodesis. However, in some cases as described, an altered femoral neck-shaft angle made the use of an intramedullary nail nearly impossible.

*Plate osteosynthesis* is the most frequently described technique in the literature on fractures in hip arthrodesis (77%). Plates were used for neck fractures, intertrochanteric fractures and subtrochanteric fractures. In our opinion, the most challenging fracture patterns are very proximal/medial fractures (neck fractures), due to their difficult close proximity to the acetabulum and limited fixation options. While single or double plating generally provides more stability, we believe that the extensive surgical wounds are less favourable for the primarily geriatric population and may increase the risk of fracture-related infections. Plate fixation is less favourable for patients with severe gluteal muscle atrophy, as the surgery requires further muscle detachment. Furthermore, we think that *patient-specific implants,* which are feasible in acetabular surgery, would be applicable in fractures of hip arthrodesis [28].

*Total hip arthroplasty* for hip arthrodesis joints could be a technically demanding and invasive procedure due to the lack of surgical landmarks secondary to ankylosis [29, 30]. However, most studies reported good to excellent results after the conversion of a hip arthrodesis to a total hip arthroplasty [29–33]. In these studies, the indications for total hip arthroplasties were mainly complaints of low back pain, knee pain and hip problems, likely due to altered biomechanics. These studies indicated relief of low back pain and knee discomfort, improved hip mobility, and correction of limb-length discrepancies postoperatively. Survival rates of the total hip arthroplasty ranged from 91% after 10 years to 70% after 30 years. However, in younger patients or those with previous surgical arthrodesis, failure rates can reach up to 33% [29–34]. We found only one study that described acute conversion to a total hip arthroplasty in a fractured hip arthrodesis. One case involved an intertrochanteric fracture previously treated with a DCS, resulting in a non-union at the same site. The other case was a subtrochanteric fracture. Both cases were successfully treated with an acute total hip arthroplasty [35]. We believe that a total hip arthroplasty could be a viable solution if the patient is fit enough to undergo a relatively invasive procedure and already has arthrodesis-related complaints.

*Overall*, the literature demonstrated a variety of implant choices for different types and levels of fractures in hip arthrodesis. Based on this, we propose a treatment algorithm presented in a flowchart for managing hip arthrodesis fractures (Fig. 11). For the most proximal/medial fractures, we recommend a tailored approach with 3D-planned cannulated screws, DHS, regular plating or patient-specific implants due to the patient-specific anatomy. The more distal fractures can be addressed with conventional surgical options, such as DHS or intramedullary nailing if the femoral neck-shaft angle allows for it. A total hip arthroplasty could be a viable solution if the patient is fit for relatively invasive surgery and has preoperative complaints related to the arthrodesis.

### Limitations

This systematic review is limited by the level of evidence available for treatment of fractured hip arthrodesis. Current recommendations for treatment are based on the interpretation of existing evidence combined with experiences from clinical practice. We acknowledge that a more solid foundation for an algorithm, supported by higher levels of evidence beyond case series would be desirable. However, the current experiences, along with the literature review, encompass all that is presently known about this injury.

## Conclusion

Acute fractures in arthrodesed hips in fragile geriatric patients can be treated minimally invasively with 3D-planned percutaneous screw fixation. This technique is most suitable for femoral neck fracture types. Alternative surgical options include DHS, intramedullary nailing, plate osteosynthesis or conversion to total hip arthroplasty, for which a treatment algorithm is provided.

## Data Availability

No datasets were generated or analysed during the current study.
